# Combining 3D printing technology with customized metal plates for the treatment of complex acetabular fractures: A retrospective study

**DOI:** 10.1371/journal.pone.0317496

**Published:** 2025-02-14

**Authors:** RongDa Xu, He Zhang, SiYu Duan, HaiRui Liang, Ming Sun, Hang Wen, HanFei Liu, XueTing Zhou, ZhenCun Cai

**Affiliations:** 1 Department of Orthopedics Surgery, Central Hospital Affiliated to Shenyang Medical College, Shenyang, Liaoning, China; 2 Sports Medicine, Tongliao People’s Hospital, Tongliao, Inner Mongolia, China; 3 Liaoning Province Key Laboratory for Phenomics of Human Ethnic Specificity and Critical Illness and Shenyang Key Laboratory for Phenomics, Shenyang Medical College, Shenyang, Liaoning, China; Southern Medical University Nanfang Hospital, CHINA

## Abstract

**Purposes:**

The purpose of this study is to evaluate the clinical outcomes of combining 3D printing technology with customized metal plates in the treatment of complex acetabular fractures.

**Methods:**

A retrospective study was conducted on 42 patients with complex acetabular fractures treated at our hospital between September 1, 2020 and May 31, 2022. The patients were divided into two groups: the conventional group and the 3D printing group, with 21 individuals in each group.The conventional group underwent surgery using traditional surgical techniques, with appropriate bending and adjustment of the metal plates during the procedure. In the 3D printing group, preoperative 3D printing technology was utilized to create a physical model of the fracture, and individualized metal plates were customized based on the model after virtual reduction. Comparison was made between the two groups of patients regarding surgical approach, operative time, instrument handling time, intraoperative blood loss, number of fluoroscopy scans, fracture healing time, quality of fracture reduction postoperatively, hip joint function at 12 months postoperatively, and occurrence of complications during the follow-up period.

**Results:**

The 3D printing group showed significantly shorter surgical time (124.76±12.89 minutes vs. 174.05±12.51 minutes), instrument operation time (44.57±5.32 minutes vs. 62.9±7.47 minutes), intraoperative blood loss (337.38±51.95 mL vs. 545.24±74.39 mL), and intraoperative fluoroscopy frequency (8.25±1.18 times vs. 10.52±1.6 times) compared to the conventional group (P<0.001). The postoperative fracture reduction quality in the 3D printing group was good in 95.24% (20/21) of cases, significantly higher than the 61.90% (13/21) in the conventional group (P = 0.02). The excellent and good hip function rate in the 3D printing group was 90.48% (19/21), which was also significantly higher than 57.14% (12/21) in the conventional group (P = 0.01). No significant difference was observed between the two groups in fracture healing time (13.95±1.07 weeks vs. 13.81±1.17 weeks) and complication rate (9.52% vs. 28.57%) (P = 0.14; P = 0.24).

**Conclusion:**

The application of 3D printing technology in conjunction with individualized customization of metal plates for the treatment of complex acetabular fractures can shorten surgical and instrument handling time, reduce intraoperative blood loss, and improve the quality of fracture reduction as well as the recovery of hip joint function.These results provide new insights and technical support for the treatment of complex acetabular fractures.

## 1. Introduction

Acetabular fractures are a severe type of fracture, typically classified into simple and complex categories. Complex acetabular fractures are further subdivided based on the Letournel-Judet classification system, including posterior wall + posterior column fractures, posterior wall + transverse fractures, T-shaped fractures, anterior column + posterior hemi-transverse fractures, and both column fractures [1]. These complex acetabular fractures typically involve various anatomical structures of the acetabulum, necessitating a more comprehensive and sophisticated treatment strategy.Treatment typically involves surgical intervention aimed at restoring the stability of the fracture and joint function. However, due to the complex anatomical structures and limited surgical approaches of acetabular fractures, traditional surgical methods may face challenges in dealing with complex acetabular fractures, potentially making it difficult to achieve precision and simplicity in surgery.

Based on the findings of our previous research, 3D printing technology is highly beneficial for the treatment of posterior wall and posterior column fractures of the acetabulum [2]. These fractures are classified as a simple type of acetabular fracture according to the Letournel-Judet classification. The primary aim of this study is to explore the therapeutic effects of 3D printing technology on this simple type of fracture. Based on these research findings, our research group has already validated the effectiveness of 3D printing technology in the treatment of simple fractures. However, there are significant differences in diagnostic and treatment strategies between simple and complex acetabular fractures. Therefore, this study further extends the research in this area. By expanding case selection, patient age, and clinical context, this study focuses on analyzing the application and effectiveness of 3D printing technology in the treatment of complex acetabular fractures classified under the Letournel-Judet system.

Currently, 3D printing technology has made significant progress in orthopedic surgery, particularly in fracture treatment, joint replacement, spinal surgery, and orthopedic oncology [3–5]. It has gradually become an indispensable technology in many complex surgeries. In the treatment of acetabular fractures, traditional surgical methods require the intraoperative adjustment of plate positioning and shaping based on the fracture reduction. This process is time-consuming and labor-intensive, and even after shaping, the plate may not fully conform to the anatomical structure of the acetabulum. This limitation can affect fracture reduction outcomes and the recovery of hip joint function [6,7]. Currently, most studies utilize 3D printing technology to create pelvic models preoperatively, which can assist doctors in gaining a more intuitive understanding of the displacement of fractures and simulate the reduction process, thereby enhancing the precision of surgery and treatment effectiveness [8–10]. However, this method still requires the use of conventional anatomical plates for surgical procedures. During surgery, if the fit between the conventional anatomical plates and the fracture site is not ideal, it may necessitate further bending of the plates, thereby adversely affecting the stable fixation of the fracture [11,12]. The application of 3D printing technology and the production of customized metal plates in fracture treatment may offer a more personalized, precise, and effective treatment approach, potentially providing better treatment outcomes for patients.

Although 3D printing technology has been widely applied in the treatment of various fractures, research on its use in the treatment of complex acetabular fractures remains relatively scarce, particularly in conjunction with custom metal plates. To address this research gap, this study proposes an innovative treatment strategy: the use of 3D printing technology to create precise fracture reduction models, combined with custom metal plates, to overcome the limitations of traditional treatment methods in terms of fracture reduction accuracy and treatment outcomes. Based on computer virtual surgery technology, our research group created physical models of the fractures after reduction. Doctors determined the shape, size, and optimal placement of the metal plates using these models, which were then specially designed by engineers through the integration of medicine and engineering, resulting in customized individualized metal plates. By comparing the effectiveness of 3D printing technology combined with customized metal plates to traditional surgical treatment for complex acetabular fractures, we aim to evaluate the advantages of their combined application in improving the treatment outcomes and patient recovery quality of complex acetabular fractures. The main hypothesis of this study is that the application of 3D printing technology combined with custom metal plates in the treatment of complex acetabular fractures can improve fracture reduction accuracy, optimize the surgical procedure, promote the healing process, and effectively enhance postoperative functional recovery and quality of life for patients.

## 2. Materials and methods

### 2.1. Participants

A retrospective analysis was conducted on patients with complex acetabular fractures admitted to the Central Hospital Affiliated to Shenyang Medical College from September 1, 2020 to May 31, 2022. Inclusion criteria are as follows: (1) Patients aged ≥18 years; (2) Complex acetabular fractures classified according to Letournel-Judet classification; (3) Fresh closed fractures requiring surgical intervention(fracture occurring within <3 weeks); (4) Availability of complete follow-up data. Exclusion criteria include: (1) Open or pathological fractures; (2) Old fractures (fracture occurring within>3 weeks); (3) Letournel-Judet classification as other types of fractures; (4) Inability to mobilize lower limbs due to reasons other than injury (e.g. neurological disorders); (5) Lost or incomplete follow-up data; (6) Poor health status or presence of other severe complications affecting postoperative rehabilitation exercises: (7) Simultaneous presence of another fracture (such as femoral neck fractures).

According to different treatment regimens, 21 patients who opted for treatment with 3D printing combined with customized plates were categorized as the 3D printing group, while the remaining 21 patients who underwent traditional surgical treatment were categorized as the conventional group. Data including gender, age, BMI, mechanism of injury, and fracture classification were recorded for both groups.

### 2.2. Ethical approval

This study has obtained approval from the Medical Ethics Committee of the Central Hospital Affiliated to Shenyang Medical College (Approval No: 2022012). All patients participating in the study were informed and consented to the use of their personal information and clinical data for research analysis. Prior to the start of the study, all patients signed a written informed consent form. The researchers provided a detailed explanation of the study’s purpose, procedures, potential risks, and the voluntary nature of participation, ensuring that patients fully understood and agreed to participate in the study.

### 2.3. 3D printing model

**Fig 1:**
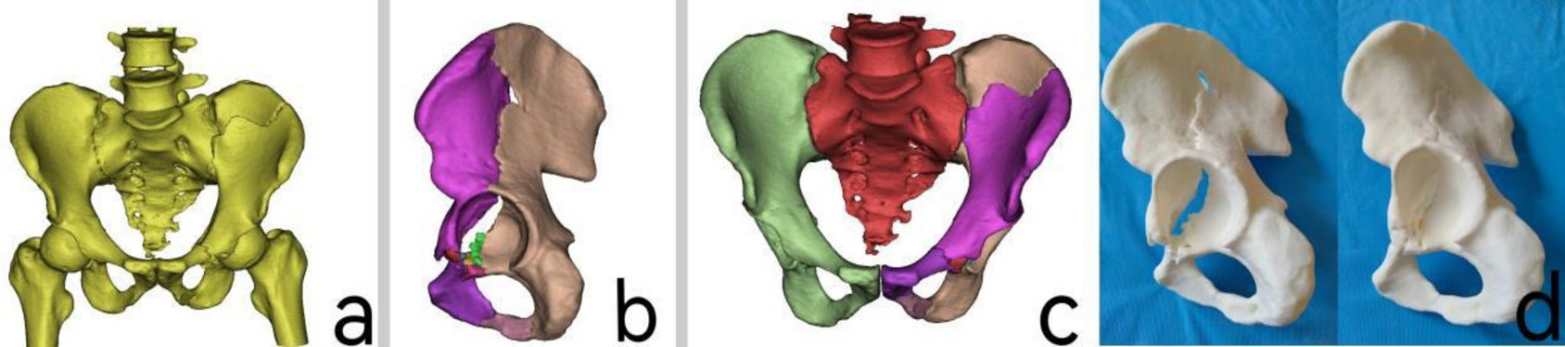
**a:** Three-dimensional modeling of the patient’s pelvis using Mimics software based on CT imaging data. **b:** Removal of the femur and lumbar vertebrae, followed by separation of the fracture fragments using the threshold selection function, resulting in the three-dimensional modeling of the patient’s pelvic fracture. **c:** Anatomical virtual reduction of the fracture fragments to obtain the three-dimensional model of the pelvis after reduction. **d:** Printing of life-sized 1:1 scale fracture physical models and fracture physical models after virtual reduction.

High-resolution CT scans were used to accurately obtain three-dimensional structural data of the surgical area. The CT data of the patient’s fracture site was imported into the Mimics 20.0 software workstation (Materialise, Belgium) in DICOM format to reconstruct the three-dimensional model of the pelvic fracture (Fig 1A). First, the bone region is initially segmented based on the gray value range of bone tissue. Then, manual segmentation tools are used to finely correct complex details to extract complete bone structure data, while removing the interference of surrounding soft tissues. Next, a smoothing tool is applied to eliminate model noise and optimize surface quality, ensuring the clarity and accuracy of the reconstructed model. During the 3D model analysis, the Mimics measurement tool is used to evaluate the fracture area in detail, including key parameters such as fracture displacement and angle changes. To better observe fracture characteristics, the femur and lumbar sections are removed, and the pelvic fracture model is examined through multi-angle rotation for a three-dimensional inspection. Then, the region segmentation tool was utilized to extract each bone fragment, implementing color staining to present them in different colors (Fig 1B). Subsequently, virtual reduction of the fracture fragments was performed using the move and rotate functions (Fig 1C). Next, the three-dimensional model of the pelvic fracture and the fracture data after virtual reduction will be exported as stereolithography (STL) format files. These files will then be imported into FlashPrint 5 software (FlashForge, China). Utilize polylactic acid (PLA) as the printing material; the standard printing temperature for PLA consumables is 215–220°C. Transmit the converted files to the 3D printer (Dreamer, China). Finally, print out physical models of the fracture, both before and after virtual reduction, at a 1:1 scale (Fig 1D).

### 2.4. Designing customized metal plates

**Fig 2:**
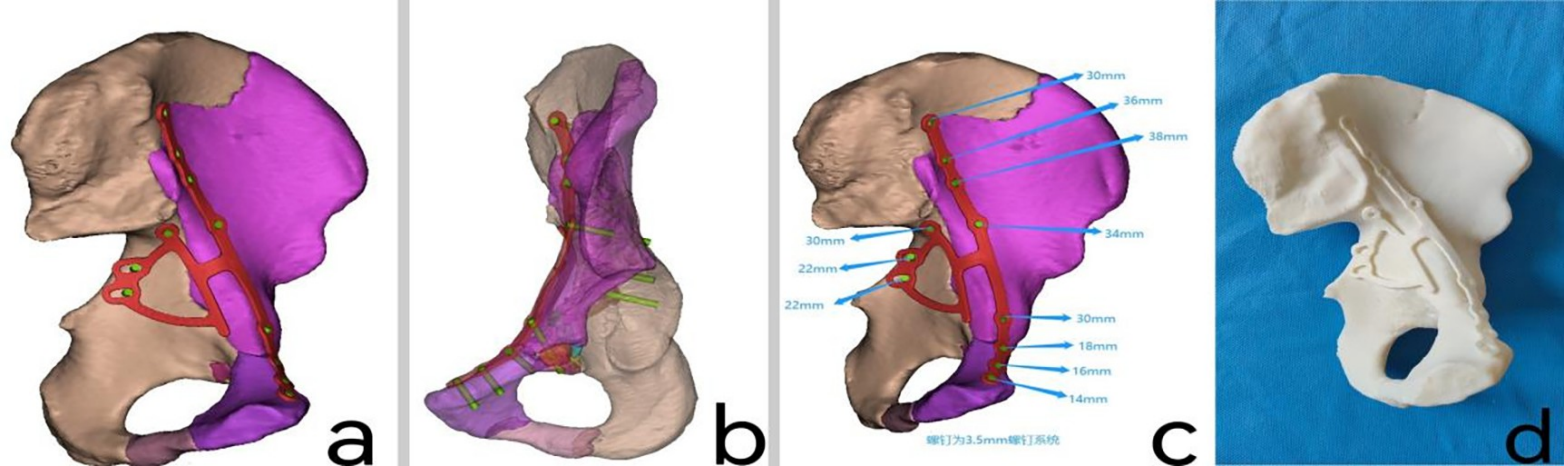
**a:** Computer simulation of the size, shape, and specific placement position of the metal plate. **b:** Computer transparency of the pelvic model to design screw lengths and observe whether the screws penetrate the acetabulum. **c:** Simulation of screw lengths through computer modeling. **d:** Customization of the metal plate model and perfect fitting at the acetabular fracture site.

To achieve stable reduction of the fracture area, detailed design of the placement, shape, and size of the customized metal plates was carried out using Unigraphics NX software (Siemens PLM Software, USA), based on the computer-aided virtual reduction of the pelvic fracture (Fig 2A). Then, using polylactic acid (PLA) as the printing material, the physical models of the metal plates were printed using FlashPrint 5 software. Subsequently, the physical models of the metal plates were imported into Mimics 20.0 software through reverse scanning. On the computer-aided virtual reduction model of the fracture, the placement of the customized metal plates was simulated once again to confirm their positioning. Next, the fracture model underwent transparency processing to confirm the implantation angle of the screws, ensuring that they do not penetrate into the joint cavity (Fig 2B). The measurement function was utilized to confirm the length of the screws, and the recommended length of each screw was marked beside each screw hole on the metal plate (Fig 2C).Finally, using pure titanium TA3 as the raw material, the metal plates were processed and shaped at the processing plant to produce authentic customized metal plates (Fig 2D). The metal plates then underwent treatments such as sandblasting, magnetic polishing, and ultrasonic cleaning before being sent to a dedicated quality inspection department for testing. After passing the tests, the metal plates were labeled and packaged. Prior to surgery, the metal plates were subjected to high-temperature and high-pressure sterilization for disinfection.

### 2.5. Preoperative preparation

After admission, patients underwent routine examinations. Preoperatively, all patients underwent pelvic anteroposterior X-rays (Netherlands, Philips digital radiography DR system) and whole pelvic CT scans with three-dimensional reconstruction (Netherlands, Philips 256-slice spiral CT scanner, scan thickness 0.6 mm). Three senior physicians classified the fractures based on the imaging data and selected patients with complex acetabular fractures according to the Letournel-Judet classification system. Detailed records of the patient’s basic information, including age, gender, BMI, and time from injury to surgery, are kept. Preoperative hemoglobin levels are also recorded, and the number of patients with coagulation dysfunction, heart dysfunction, pulmonary dysfunction, and other organ dysfunctions are statistically analyzed. General anesthesia was administered to all patients, and on the day before surgery, they underwent procedures such as skin preparation, enema, and urinary catheterization.

### 2.6. Surgical procedure

The patient undergoes general anesthesia in an appropriate position. The surgical approach, including the ilioinguinal approach, modified Stoppa approach, para-rectus abdominis approach, and Kocher-Langenbeck approach, is chosen by the surgeon based on the fracture pattern and the type of metal plates used. All surgeries in this study were performed by the same team of experienced surgeons. This ensures that the skill level and experience of the surgeons remained consistent, minimizing the impact of operator variability on the surgical outcomes.

In the 3D printing group, doctors use Mimics software to perform three-dimensional reconstruction of the patient’s CT data. Through 3D printing technology, a life-sized model of the patient’s pelvis and fracture site is created for preoperative simulation. During the surgery, the doctor first accurately repositions the fracture fragments according to the 3D printed model and compares the alignment with the physical model to confirm the accuracy of the reduction. Subsequently, according to the preoperative personalized design plan, the custom metal plate, which has been pre-designed and processed, is fitted onto the repositioned fracture site and fixed in place with titanium alloy screws. The custom metal plate used is made of titanium alloy, which offers excellent biocompatibility and mechanical strength. In the conventional group, fracture reduction is performed based on the surgeon’s experience, relying on visual observation and palpation during the surgery. After reducing the fracture, the surgeon selects a standardized anatomical plate, which is then bent or cut according to the reduced fracture’s condition. The plate is subsequently fixed in place using standardized screws.

Regardless of whether it’s the 3D printing group or the conventional group, intraoperatively, screw lengths are measured, and fluoroscopy from multiple angles is used to ensure that the screws do not penetrate into the joint cavity. Furthermore, various directions of hip joint movement are tested to observe the stability of the fracture, ensuring the success of the surgery. Drainage tubes are placed, and the incisions are closed layer by layer. Surgical approach, operative time, number of intraoperative fluoroscopy scans, intraoperative blood loss, and other data are recorded for both groups.

### 2.7. Postoperative management

Due to the presence of implanted metal plates, the postoperative CT images exhibit significant metallic artifacts, which have compromised the accuracy of image interpretation. Therefore, we have opted to utilize X-rays to assess the quality of fracture reduction. On the first day postoperatively, both groups of patients underwent follow-up pelvic X-rays in anterior-posterior, inlet, and outlet views. Drainage tubes were removed based on the drainage volume. Sutures were removed as appropriate at 2 weeks postoperatively. Ankle pump exercises were initiated 6 hours after surgery, followed by quadriceps femoris contraction exercises 2–3 days after surgery, hip joint flexion and extension exercises at 2 weeks postoperatively, and partial weight-bearing exercises at 6 weeks postoperatively. During the follow-up period, pelvic X-ray examinations were performed monthly for the first two months postoperatively, followed by weekly examinations until the fracture healed completely. According to imaging assessments during follow-up, the fracture healing status was evaluated. X-rays revealed indistinct fracture lines with continuous callus formation traversing the fracture lines, indicating fracture union. The follow-up period was not less than 12 months.

### 2.8. Assessment of therapeutic efficacy

Comparison of general patient characteristics between the two groups, including age, gender, BMI, mechanism of injury, fracture classification, preoperative preparation time, total hospitalization time, and average follow-up time, where preoperative preparation time is defined as the time from patient admission to the start of surgery. Comparison of surgical approaches between the two groups, with single surgical approach defined as completing the surgery using only one approach, and combined surgical approach defined as completing the surgery using two or more approaches. Comparison of surgical time, instrument operation time, intraoperative blood loss, intraoperative fluoroscopy times, and fracture healing time between the two groups. Surgical time is defined as the duration from incision to wound closure. Instrument operation time is defined as the time from adjusting the position of the metal plates to screwing all screws into the plates. Intraoperative blood loss is calculated by measuring the volume of irrigation fluid, blood in suction bottles, and blood on gauze. Intraoperative fluoroscopy times refer to the total number of fluoroscopic examinations during surgery. Fracture healing time is recorded from the first day after surgery until the patient meets the diagnostic criteria for fracture healing. Radiological assessment was performed by three experienced orthopedic surgeons, and the criteria for evaluating the quality of fracture reduction are as follows: displacement of <2mm is considered good, while displacement of ≥2mm is considered fair. At 12 months postoperatively, hip joint function was assessed based on the Harris score [13], with specific criteria as follows: hip joint function was considered excellent/good (Harris score ≥80) or fair/poor (Harris score <80). Complications during the follow-up period for both groups of patients were recorded, including inflammatory reactions, heterotopic ossification, infection, iatrogenic neurological symptoms, traumatic arthritis, etc.

### 2.9. Statistical analysis

Statistical analysis was performed using SPSS 27.0 statistical software(IBM, Armonk, NY, USA). Categorical data were expressed as frequencies or percentages, and the chi-square test was used for group comparisons. For continuous data, the mean ± standard deviation was used to represent the data. Before analyzing continuous data, the Shapiro-Wilk test (used for small sample size data to test normality) was conducted to assess whether the data followed a normal distribution. For continuous variables that followed a normal distribution, independent samples t-test was used for group comparisons (suitable for comparing two independent groups where data in each group are normally distributed and pass the homogeneity of variance test). For data that did not follow a normal distribution, the Mann-Whitney U test was used for non-parametric analysis (suitable for comparing distribution differences between two independent samples, especially when data do not meet the normality or homogeneity of variance assumptions). A P-value of less than 0.05 was considered statistically significant.

## 3. Results

### 3.1. Baseline data

A total of 42 patients were included in this study, with 21 patients in both the conventional group and the 3D printing group. There were no statistically significant differences between the two groups in terms of patient gender, age, BMI, mechanism of injury, and type of fracture (P > 0.05). The preoperative preparation time and total length of hospital stay were longer in the 3D printing group compared to the conventional group, but the differences were not statistically significant (P > 0.05). The average follow-up time for both groups of patients was at least 12 months (Table 1).

**Table 1:** Baseline characteristics between two groups of patients.

	Conventional group (n = 21)	3D printing group(n = 21)	P value
Age (years)	41.1±11.59	43.81±10.6	0.37
Sex (n)			0.51
Male	15	13	
Female	6	8	
BMI (kg/m^2^)	24.43±3.03	24.86±3.25	0.66
Mechanism of injury (n)			0.57
Fall from height injury	4	7	
Traffic accident injury	10	8	
Other injuries	7	6	
Types of fractures (n)			0.95
Posterior wall + posterior column fractures	8	10	
Posterior wall + transverse fractures	4	3	
T-shaped fractures	5	5	
Anterior column + posterior hemi-transverse fractures	2	2	
Both column fractures	2	1	
Preoperative preparation time (days)	5.95±0.81	6.48±1.03	0.08
Hospital stay (days)	11.24±1.73	11.48±1.72	0.66
Follow-up time (months)	13.05±1.0	13.19±1.1	0.75

### 3.2. Preoperative examination results

The average hemoglobin level in the 3D printing group was (88.4±14.4) g/L, and the average hemoglobin level in the traditional group was (87.5±15.2) g/L. The difference was not statistically significant (P = 0.84). In the comparison of coagulation function, cardiac function, pulmonary function, and abnormalities in other organ functions, there were no statistically significant differences between the two groups (P = 1.0; P = 0.70; P = 0.50; P = 0.47, respectively) (Table 2).

**Table 2:** Comparative analysis of preoperative examination results.

	Conventional group (n = 21)	3D printing group(n = 21)	P value
hemoglobin (g/L)	87.5±15.2	88.4±14.4	0.84
coagulation function (n)			1.0
abnormal	4	3	
normal	17	18	
cardiac function (n)			0.70
Abnormal	3	5	
Normal	18	16	
pulmonary function (n)			0.50
Abnormal	7	5	
normal	14	16	
other organ functions (n)			0.47
abnormal	6	4	
normal	15	17	

### 3.3. Surgical outcomes

**Fig 3:**
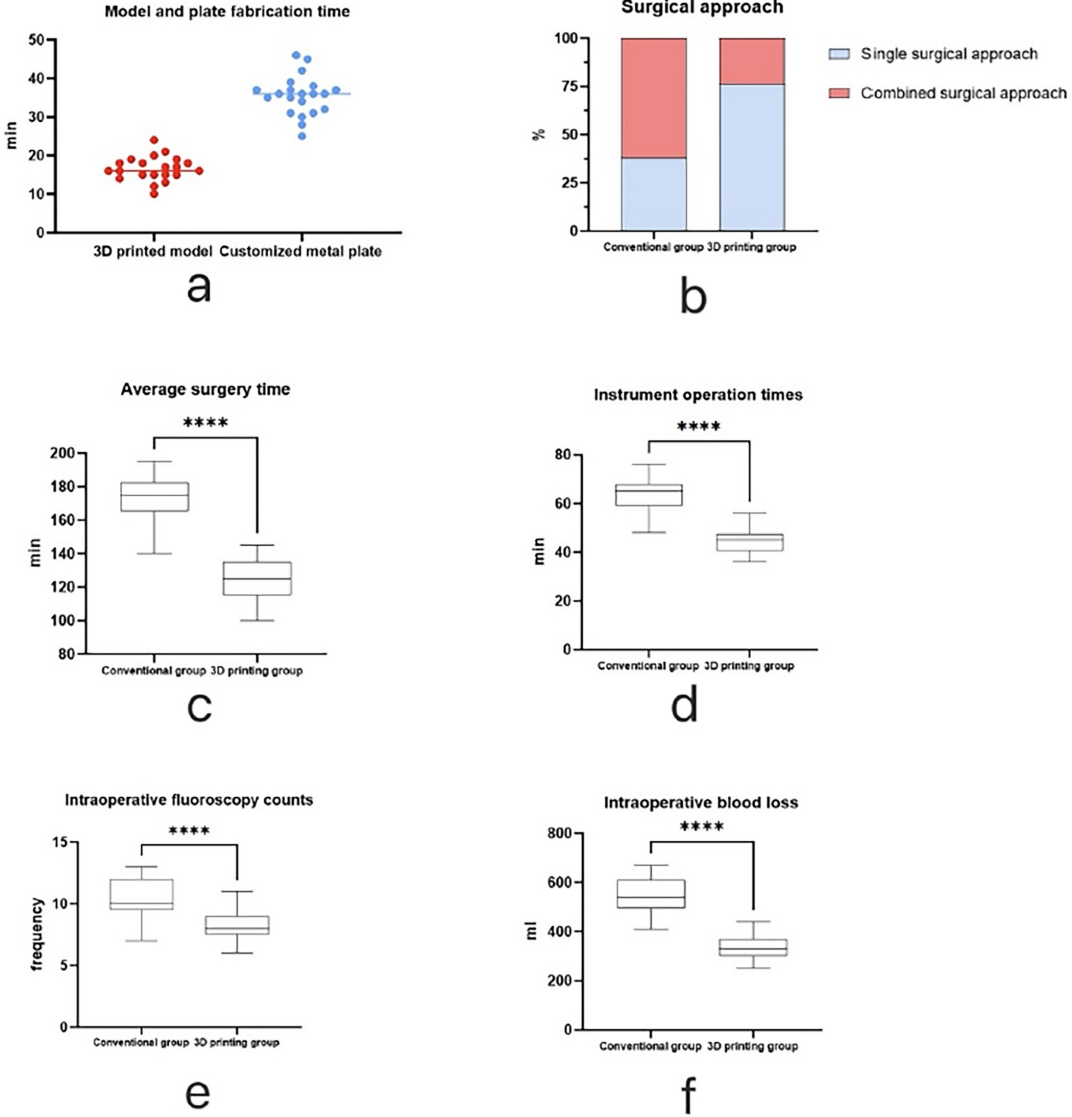
**a:** The production time of 3D printing models and customized steel plates in the 3D printing group. **b:** The percentage of patients in both groups with single or combined surgical approaches. **c-f:** The average surgical time, instrument operation time, X-ray fluoroscopy counts, and intraoperative blood loss in both groups (**** P < 0.001; ** P < 0.01; * P < 0.05; ns P > 0.05).

The average time for 3D printing group to produce physical models of fractures was 16.57±3.16 hours, while the average time for custom-made metal plates was 35.52±5.11 hours. The proportion of surgeries completed through a single surgical approach was higher in the 3D printing group compared to the conventional group, and the difference was statistically significant (P = 0.01). The average surgical time for patients in the 3D printing group was 124.76±12.89 minutes, which was less than the conventional group’s 174.05 ± 12.51 minutes. The instrument operation time in the 3D printing group was 44.57±5.32 minutes, less than the conventional group’s 62.9±7.47 minutes. The intraoperative blood loss in the 3D printing group was 337.38±51.95 ml, less than the conventional group’s 545.24 ± 74.39 ml. The number of fluoroscopy times during surgery was 8.25±1.18 times in the 3D printing group, less than the conventional group’s 10.52±1.6 times, and all differences were statistically significant (P < 0.001) (Table 3, Fig 3).

**Table 3:** Comparison of surgical outcomes between two groups of patients.

	Conventional group (n = 21)	3D printing group(n = 21)	P value
Printing time for 3D models (h)	-	16.57±3.16	-
Customizing metal plate time (h)	-	35.52±5.11	-
Surgical approach (n)			0.01
Single surgical approach	8	16	
Combined surgical approach	13	5	
Surgical time (min)	174.05±12.51	124.76±12.89	<0.001
instrument operation time (min)	62.9±7.47	44.57±5.32	<0.001
intraoperative blood loss (ml)	545.24±74.39	337.38±51.95	<0.001
intraoperative fluoroscopy counts (frequency)	10.52±1.6	8.25±1.18	<0.001

### 3.4. Postoperative follow-up data

**Fig 4:**
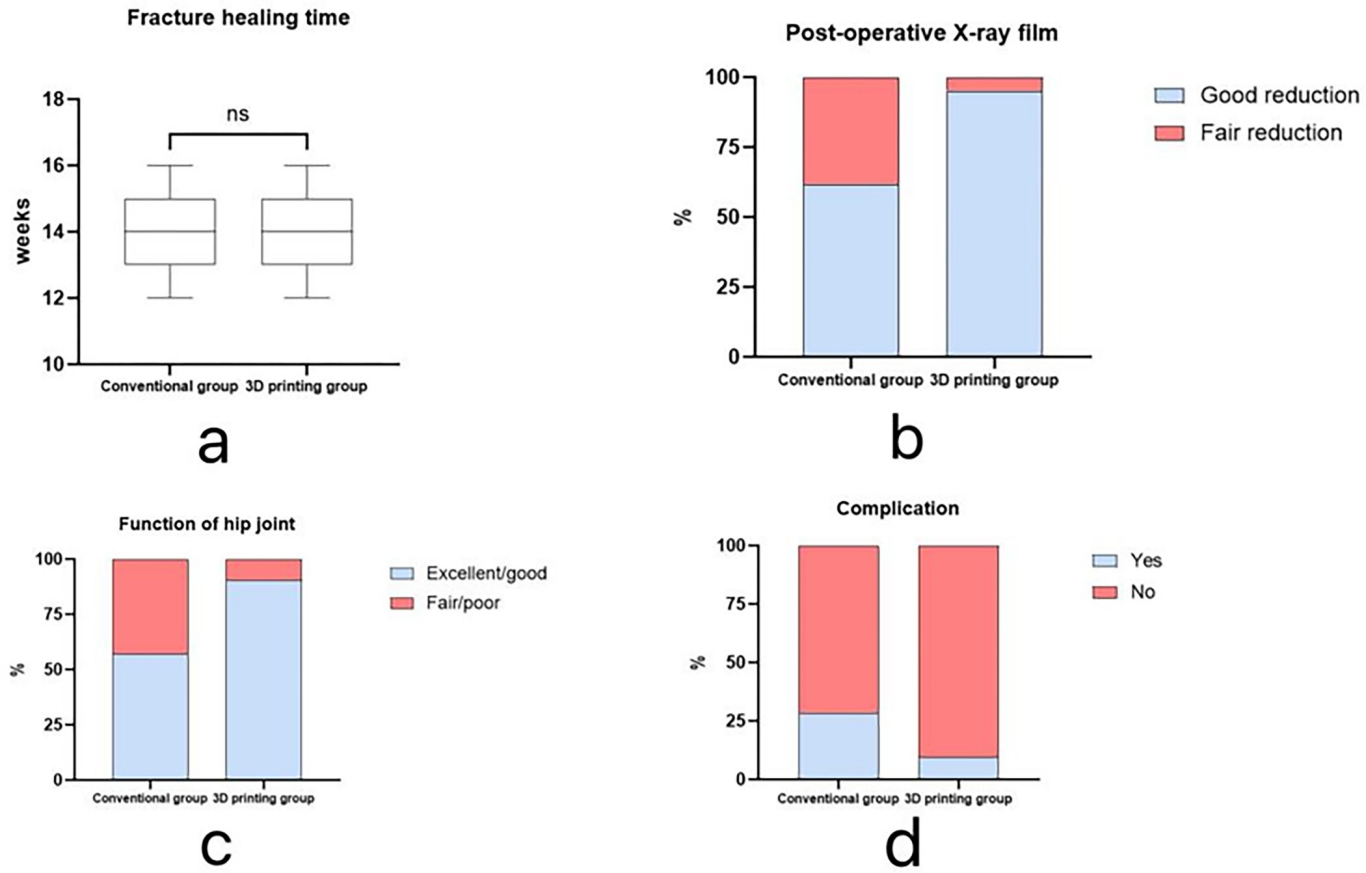
**a:** Comparison of fracture healing time between the two groups. **b:** Percentage of patients in each group with good fracture reduction quality on the first postoperative day. **c:** Percentage of patients in each group with excellent/good pelvic function at 12 months postoperatively. **d:** Comparison of overall postoperative complication rates between the two groups (**** P<0.001; ** P<0.01; * P<0.05; ns P>0.05).

**Fig 5:**
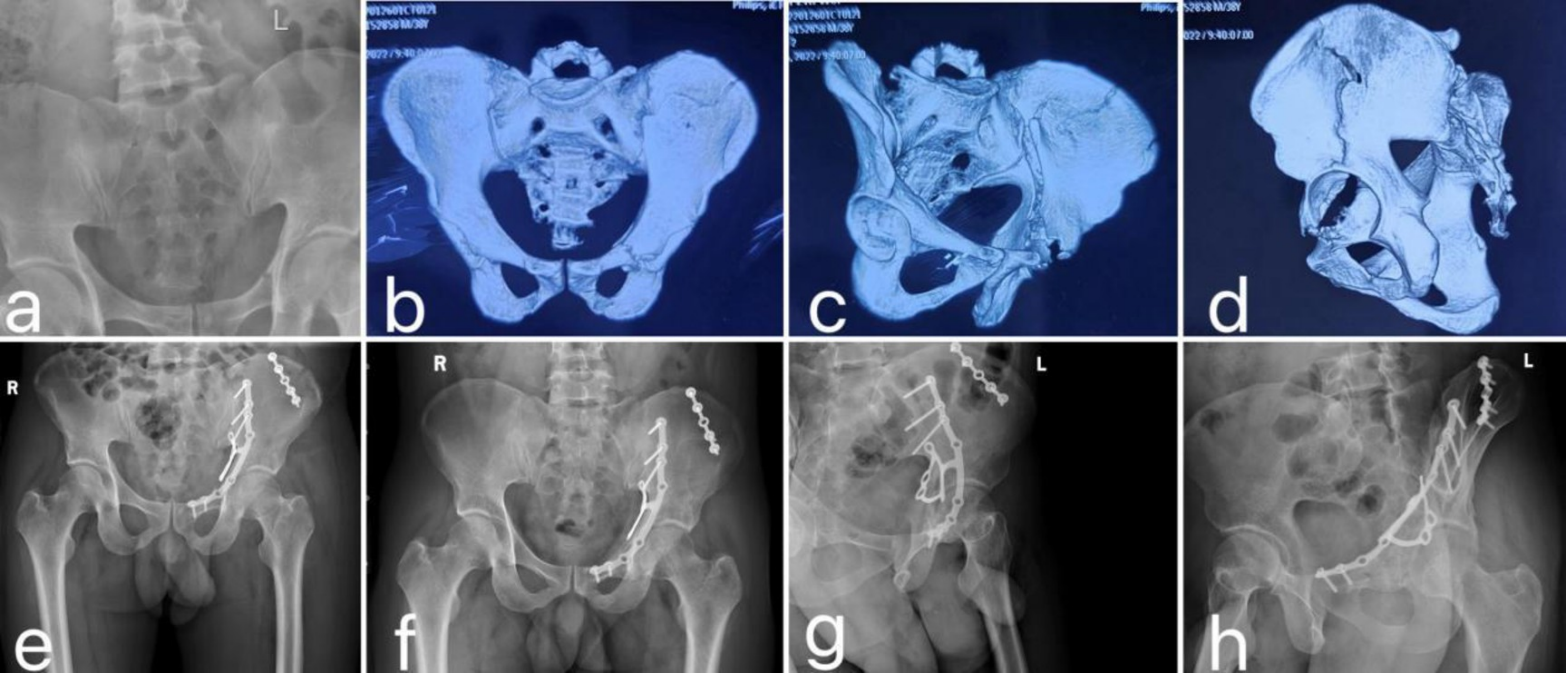
Typical Case 1: A 38-year-old male patient with a T-type acetabular fracture undergoing surgery using 3D printing technology combined with customized steel plates. **a:** Preoperative pelvic X-ray. **b-d:** Preoperative pelvic CT scan with three-dimensional reconstruction examination. **e:** Pelvic X-ray on the first day postoperatively. **f-h:** Pelvic X-rays at 12-month follow-up (including anteroposterior view, pelvic outlet oblique view, and iliac oblique view).

**Fig 6:**
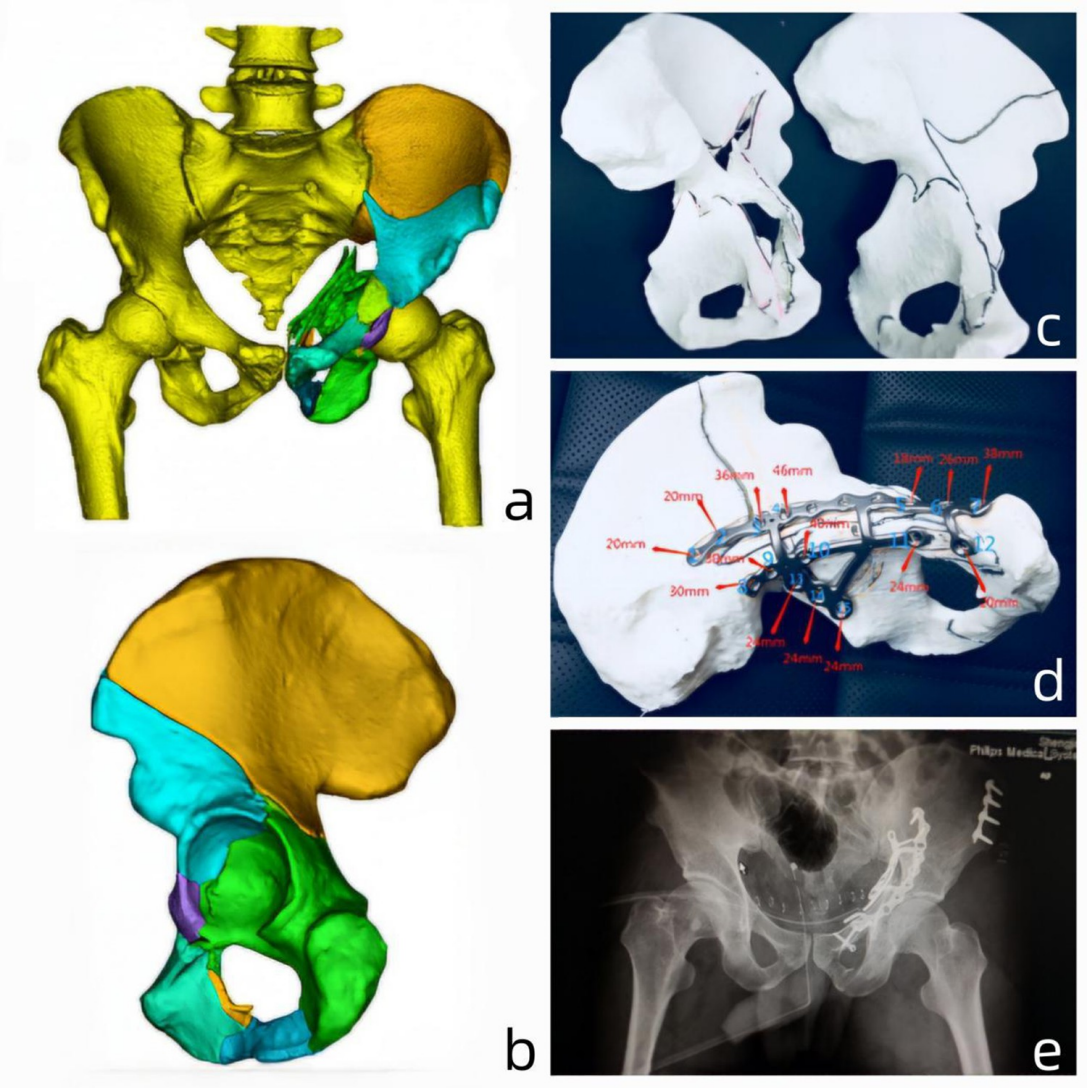
Typical Case 2: A 46-year-old female patient with both column fractures underwent surgery using 3D printing technology combined with customized metal plates. **a:** Application of Mimics software to create a three-dimensional model of the patient’s CT imaging data. **b:** Generation of a three-dimensional model of the acetabular fracture after reduction using computer-simulated surgical techniques. **c:** 3D-printed physical model of the fracture, along with a computer-simulated model after reduction. **d:** Production of customized metal plates based on the reduced fracture model, with preoperative determination of plate placement, angle, and screw length. **e:** Postoperative pelvic X-ray images.

**Fig 7:**
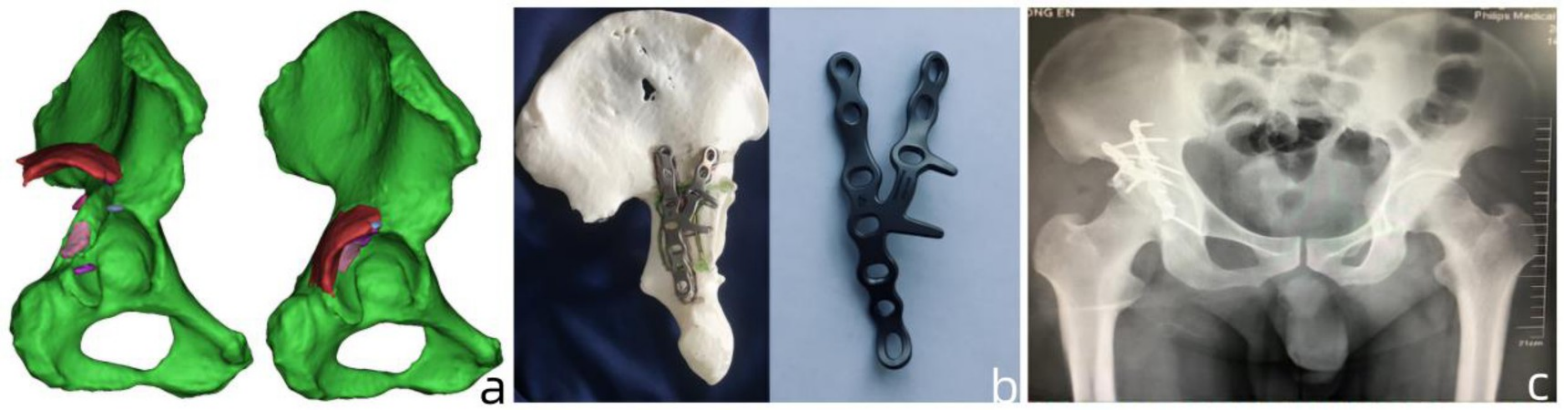
Typical Case 3: A 42-year-old male patient with Posterior wall + posterior column fractures underwent surgery using 3D printing technology combined with customized metal plates. **a:** Application of Mimics software to create a three-dimensional model of the patient’s CT imaging data. **b:** Generation of a three-dimensional model of the acetabular fracture after reduction using computer-simulated surgical techniques, followed by the production of customized metal plates with preoperative determination of plate placement and angle. **c:** Postoperative pelvic X-ray images.

The healing time of fractures in the 3D printing group (13.95 ± 1.07 weeks) was slightly longer than that in the conventional group (13.81 ± 1.17 weeks), but the difference was not statistically significant (P = 0.14). According to X-ray evaluation of fracture reduction quality on the first postoperative day and Harris evaluation criteria at 12 months postoperatively, the fracture reduction quality and hip joint function in the 3D printing group were significantly better than those in the conventional method group (good fracture reduction rate: 95.24% vs. 61.9%; excellent/good hip joint function rate: 90.48% vs. 57.14%), and all differences were statistically significant (P = 0.02; P = 0.01).During the postoperative follow-up, there were 2 cases of traumatic arthritis, 2 cases of infection, 1 case of heterotopic ossification, and 1 case of inflammatory reaction in the conventional group, totaling 6 cases. In the 3D printing group, 1 patient developed an inflammatory reaction, and 1 patient developed heterotopic ossification 2 months postoperatively, totaling 2 cases.The number of postoperative complications in the 3D printing group was fewer than that in the conventional group, but the difference was not statistically significant (P = 0.24) (Table 4, Fig 4).The images of the patient in the 3D printing group are shown in Figs 5–7.

**Table 4:** Postoperative follow-up data for patients in two groups.

	Conventional group	3D printing group	P value
	(n = 21)	(n = 21)	
Fracture healing time (weeks)	13.81±1.17	13.95±1.07	0.14
Post-operative X-ray film, n (%)			0.02
Good reduction (<2 mm displacement)	13 (61.9)	20 (95.24)	
Fair reduction (≥2 mm displacement)	8 (38.1)	1 (4.76)	
Function of hip joint, n (%)			0.01
Excellent/good (Harris score ≥80 points)	12 (57.14)	19 (90.48)	
Fair/poor (Harris score <80 points)	9 (42.86)	2 (9.52)	
Complication, n (%)			0.24
NO	15 (71.43)	19 (90.48)	
Yes	6 (28.57)	2 (9.52)	

## 4. Discussion

In recent years, computer simulation surgery techniques and 3D printing technology have been applied in many complex fracture surgeries, allowing surgeons to achieve more reliable fracture fixation tailored to the specific conditions of the patient.Ansari et al. [14] compared two surgical approaches, traditional surgery and the use of pre-bent metal plates with 3D printing technology, in the treatment of complex acetabular fractures. The results showed that 3D printing facilitated a better understanding of the anatomical structure of acetabular fractures, leading to reduced surgical time, intraoperative blood loss, and the number of intraoperative fluoroscopy exposures. Hsu et al. [15] achieved improved outcomes in terms of surgical time and effectiveness for treating acetabular fractures by employing preoperative computer-assisted virtual reduction and intraoperative combined use of 3D-printed models and pre-bent metal plates. Compared to other studies that apply 3D printing technology for the treatment of acetabular fractures, the innovation of this research lies in the combination of 3D printing with personalized custom plates, providing a precise treatment plan for complex acetabular fractures. Many 3D printing studies primarily focus on constructing virtual models of fractures to assist with preoperative planning and design, but few studies have directly combined 3D printing technology with custom plates for clinical applications. However, for complex acetabular fractures, due to the intricate nature of the fracture site and variations between individuals, even with the use of pre-bent metal plates based on 3D-printed models, achieving an ideal fit between the plate and the acetabular anatomy may be challenging during surgery. Further bending of the plates and fixation may be required intraoperatively.

Our research team utilized 3D printing technology in conjunction with customized plates for the treatment of complex acetabular fractures. We found no statistically significant differences in preoperative preparation time and total hospital stay between the 3D printing group and the conventional group (P > 0.05). This indicates that preoperative printing of 3D models and customization of plates did not lead to a prolongation of the corresponding time, which is consistent with the findings of Hung et al. [16]. The 3D printing group demonstrated significantly lower average surgical times compared to the traditional group (P<0.001). This difference may be attributed to the personalized surgical planning provided by the 3D printing group, along with advantages such as preoperative simulation of the surgical procedure, thereby reducing unnecessary steps during surgery. This also resulted in a significant reduction in intraoperative fluoroscopy exposures for the 3D printing group (P<0.001).In addition, compared to the traditional group, the use of individualized custom plates for fracture fixation intraoperatively in the 3D printing group reduced the time spent on bending and shaping the plates, thereby significantly decreasing the instrument manipulation time (P<0.001). It is worth noting that, due to the individualized customization of plates in the 3D printing group based on the type of fracture, some customized plates may have different shapes or larger volumes. These factors may to some extent increase the surgical time and instrument manipulation time. However, overall, the 3D printing group still demonstrated shorter average surgical and instrument operation times. Utilizing 3D printing technology, surgeons gain a deeper understanding of the specific patterns of complex acetabular fractures. Consequently, they can precisely select a single, less extensive, or limited invasive surgical exposure method. This approach maximally preserves surrounding tissues and enables effective fracture fixation. As a result, the 3D printing group exhibited significantly lower proportions of single surgical approaches and intraoperative blood loss compared to the traditional group (P = 0.01; P<0.001). The aforementioned results are consistent with many studies utilizing pre-bent metal plates through 3D printing technology for acetabular fracture surgeries [17–20]. Therefore, employing 3D printing technology in conjunction with customized plates for treating complex acetabular fractures can significantly and effectively reduce surgical complexity, shorten surgical duration, and decrease intraoperative blood loss.

The 3D printing group also demonstrated superior fracture reduction quality on the first postoperative day and better hip joint functional scores at the 12-month follow-up compared to the conventional group (P = 0.02; P = 0.01). In the 3D printing group, the proportions of fractures with good reduction scores and hip joint functional scores reaching excellent or good levels were 95.24% and 90.48%, respectively, which were higher than those in the conventional group, which were 61.9% and 57.14%, respectively. These study results demonstrate that combining 3D printing models with customized plates not only effectively improves surgical efficiency and reduces surgical complexity but also significantly enhances the quality of fracture reduction and improves the postoperative recovery of hip joint function. Additionally, there were no significant differences between the two groups in terms of fracture healing time and overall complication rates (P = 0.14; P = 0.24).This result has significant implications for clinical practice: first, shortening the surgical time and reducing intraoperative blood loss are crucial for the patient’s surgical tolerance and postoperative recovery; second, the improvement in fracture reduction quality and the restoration of hip joint function have a significant positive impact on the patient’s long-term quality of life. It should be noted that these studies have not yet been widely applied in clinical practice. Therefore, although these preliminary research results confirm the potential advantages of personalized customized plates in the treatment of acetabular fractures, further research and clinical validation are needed to determine their effectiveness and feasibility in actual clinical practice.

However, the combination of 3D printing technology with personalized customized plates also has some drawbacks. Firstly, both 3D models and customized plates incur higher costs, with specific prices depending on factors such as materials used and printing size [21]. Secondly, 3D printing technology cannot accurately reflect the surrounding soft tissues, blood vessels, nerves, etc., of the fracture site [22]. Thirdly, although 3D printing is a rapid prototyping technology, it still requires a considerable amount of time to complete.

A limitation of this study is that complex acetabular fractures are relatively rare compared to other fractures, resulting in a small sample size. Therefore, larger studies are needed in the future to confirm the effectiveness of combining 3D printing technology with customized plates in the surgical treatment of complex acetabular fractures.

With the continuous advancement of 3D printing technology, personalized treatment plans combining custom plates are expected to become the standard approach for treating complex acetabular fractures. Therefore, future research should not only focus on the application of this technology in complex acetabular fractures but also explore its potential in other types of complex fractures. Through these studies, the goal is to provide more precise and safer treatment options for clinical practice, promoting the development of personalized medicine.

The limitations of this study are mainly reflected in the following aspects. First, as a retrospective design, the data may be affected by patient selection bias and inconsistent treatment standards, which could impact the accuracy of the results. Second, due to the relatively rare occurrence of complex acetabular fractures, the sample size in this study is small, limiting the statistical power of the results and the ability to generalize them to broader clinical practice. To further validate the effectiveness of 3D printing technology combined with custom plates in the surgical treatment of complex acetabular fractures, future research should expand the sample size, particularly through multicenter, large-scale prospective clinical trials to ensure the comprehensiveness and reliability of the data.

## 5. Conclusion

The application of 3D printing technology combined with personalized customized metal plates in the treatment of complex acetabular fractures can shorten surgical and instrument operation time, reduce intraoperative blood loss, and improve the quality of fracture reduction and recovery of hip joint function. In future clinical practice, especially in the treatment of complex acetabular fractures, 3D printing technology could be considered for preoperative planning. It allows for the precise design of metal plates based on the patient’s individual anatomical structure, enabling personalized treatment and optimizing surgical outcomes.

## Supporting information

S1 Data (XLSX)
